# Occupational Organophosphorus Pesticide Exposure and Metabolic Syndrome: Implications of PPARγ

**DOI:** 10.3390/toxics14090788

**Published:** 2026-09-06

**Authors:** Samar Sakr, Mai M. Eldaly, Raghda Ali Elshamy, Noura Almadani, Hanaa A. Nofal, Sherif Attia Hammad, Mamdouh Eldesoqui, Wafaa Ibrahim Soliman

**Affiliations:** 1 Forensic Medicine and Clinical Toxicology Department, Faculty of Medicine, Zagazig University, Zagazig 44519, Egypt; 2 Medical Biochemistry and Molecular Biology Department, Faculty of Medicine, Zagazig University, Zagazig 44519, Egypt; 3 Public Health and Community Medicine Department, Faculty of Medicine, Zagazig University, Zagazig 44519, Egypt; 4 Department of Community and Psychiatric Mental Health Nursing, College of Nursing, Princess Nourah bint Abdulrahman University, P.O. Box 84428, Riyadh 11671, Saudi Arabia; 5 Kuwait Poison Control Center, AL-Amiri Hospital, Shark, Kuwait City 15303, Kuwait; 6 Department of Basic Medical Sciences, College of Medicine, AlMaarefa University, Riyadh 13713, Saudi Arabia

**Keywords:** OPPs, MS, farm workers, *PPARγ*, PON1

## Abstract

Organophosphorus pesticides (OPPs) are widely used and have recently been linked to metabolic syndrome (MS). This study aimed to investigate the probable association between chronic OPP exposure and MS among farm workers in Sharkia Governorate, Egypt, and to assess the potential role of *peroxisome proliferator-activated receptor gamma (PPARγ*). This comparative cross-sectional study included 140 participants, equally divided into OPP-exposed farm workers and non-exposed subjects. OPP exposure was confirmed by detecting plasma residues and cholinesterase activity. MS was diagnosed by assessing body mass index (BMI), waist circumference (WC), blood pressure, plasma glucose, serum insulin, and lipid parameters. Oxidative and inflammatory markers, including malondialdehyde (MDA), gamma-glutamyl transferase (GGT), ferritin, superoxide dismutase (SOD), tumor necrosis factor-alpha (TNF-α), and high-sensitivity C-reactive protein (hs-CRP), were measured. The mRNA expression of the *PPARγ* and *paraoxonase 1* (*PON1*) genes was also investigated. In total, 60% of farm workers demonstrated MS, compared with 10% of non-exposed participants. Workers exhibited elevated oxidative and inflammatory indices and reduced *PPARγ* and *PON1* expression. *PPARγ* positively correlated with high-density lipoprotein (HDL), SOD, and *PON1*, while negatively correlating with glucose, insulin resistance (IR), low-density lipoprotein (LDL), triglycerides (TGs), MDA, GGT, ferritin, TNF-α, and hs-CRP. The study concluded that chronic OPP exposure was associated with increased oxidative stress and inflammation, reduced *PPARγ* and *PON1* expression, disturbed glucose and lipid metabolism, and increased IR. The observed associations between *PPARγ* downregulation, metabolic disturbances, and oxidative and inflammatory markers suggest that *PPARγ* dysregulation may represent a potential mechanistic link between chronic OPP exposure and MS. However, this proposed mechanism requires further validation.

## 1. Introduction

Organophosphorus pesticides (OPPs) are widely used, accounting for 50 percent of global utilization. Significant evidence has linked long-term exposure to OPPs with metabolic dysregulations [1]. Critical among them is metabolic syndrome (MS), which affects approximately 25% of the world’s population and contributes to diabetes, cardiovascular diseases, and increased mortality rates [2].

Metabolic syndrome denotes a constellation of interrelated metabolic disorders and is clinically diagnosed depending on the presence of at least three of the metabolic dysregulations, including hypertension, hyperglycemia, insulin resistance (IR), obesity, and dyslipidemia [1].

Prolonged contact with OPPs has been reported to elevate various metabolic markers, including glucose, insulin, homeostatic model assessment for insulin resistance (HOMA-IR), glycated hemoglobin (HbA1c), cholesterol, and low-density lipoprotein (LDL). The biological mechanisms linking chronic OPPs exposure to MS are yet to be fully understood. However, oxidative stress and inflammation are widely recognized as the main players in MS [2,3].

*PPARγ (peroxisome proliferator-activated receptor gamma*) acts as a prime modulator of metabolic homeostasis by adjusting insulin sensitivity and directing fat and glucose metabolism [4]. In addition, *PPARγ* exhibits antioxidant and anti-inflammatory roles via regulating the nuclear factor erythroid 2-related factor 2 (Nrf2) [5], and suppressing the nuclear factor kappa-light-chain-enhancer of activated B cells (NF-κB) pathway [6]. A strong association has been recognized between *PPARγ* dysregulation and MS comorbidities, like obesity, IR, hypertension, and dyslipidemia [7].

The *paraoxonase 1 (PON1)* gene determines individuals’ susceptibility to OPP compounds, especially diazinon and chlorpyrifos, and reduced *PON1* activity amplifies the adverse health effects of OPP exposure. Additionally, *PON1* contributes to antioxidant and anti-inflammatory defense, and its deficiency enhances both oxidative stress and systemic inflammation [8].

*PON1* and PPAR-γ are functionally interconnected, with PPAR-γ acting as the principal regulator of *PON1* expression, particularly in the liver. This regulatory interaction contributes to antioxidant and anti-inflammatory activities, thereby helping to protect against cardiovascular disorders and improve lipid metabolism [9].

OPPs are extensively utilized in Egypt in farming as pesticides and insecticides. MS predisposes to critical diseases, including cardiovascular disorders, diabetes, and liver diseases, representing a great burden in our country. Thus, this study aimed to investigate the probable association between chronic OPP exposure and MS among farm workers in Sharkia Governorate, Egypt, and to assess the potential role of *PPARγ*.

## 2. Subjects and Methods

### 2.1. Study Design

A comparative cross-sectional study was conducted in four agricultural villages in the San Elhagar and Awlad Sakr districts of Sharkia Governorate, Egypt, between December 2024 and March 2025. A multistage random sampling technique was employed to select the study setting. In the first stage, Sharkia Governorate was selected randomly from the 29 governorates of Egypt using a simple random sampling technique. In the second stage, two districts from Sharkia (San Elhagar and Awlad Sakr districts) were selected randomly. In the third stage, two villages were randomly selected from each district by a simple random technique, yielding a total of four agricultural villages. Eligible participants were subsequently recruited from the selected villages according to the predefined inclusion and exclusion criteria. All experimental procedures and the study design were conducted in accordance with the Declaration of Helsinki and approved by the Institutional Review Board of the Faculty of Medicine, Zagazig University (IRB approval no. 787/17-11-2024).

#### 2.1.1. Sample Size

Assuming that the mean ± standard deviation (SD) of cholesterol levels among OPP occupationally exposed workers is 260 ± 99 mg/dL, and in the non-exposed group is 200 ± 149 mg/dL [1]. So, the sample size was 140 (70 per group), using OpenEpi Version 3, at CI 95%, and the power of the test was 80%.

#### 2.1.2. Participant Selection

The study included two groups: the occupational OPPs-exposed group (70 farm workers) and the non-exposed group (70 non-exposed subjects). All participants received a full explanation of both the study’s aims and methods before signing written consent. 140 subjects were included in this study after meeting the inclusion criteria.

▪ Inclusion criteria: The occupationally exposed group included male farm workers (farmers, blenders, sprayers, supervisors, and collectors), aged 18–60 years, with a history of contact with OPPs for 10 years or more in cotton farming areas where OPPs were applied.

The non-exposed group included workers involved in packing and transportation, tractor drivers, and livestock workers from the same communities with no risk of exposure at their current occupation, nor had a past occupational history of exposure to OPPs, or a previous history of acute OPP intoxication.

▪ Exclusion criteria: age data below 18 or more than 60 years, current treatment of diabetes, high blood pressure, thyroid disorders, chronic heart, renal, or liver diseases, corticosteroid treatment, and refusal to participate were the exclusion criteria applied to both groups. Farm workers with a history of occupational co-exposure to pesticides other than OPP, or previous acute OPP poisoning, were also excluded.

### 2.2. Operational Design

Sociodemographic (age) and anthropometric (BMI and WC) data were determined. Meanwhile, occupational data (types of OPPs, duration of exposure to OPPs, hours of spraying per week, precautionary equipment usage), and smoking were collected from farm workers by our trained interviewers via a questionnaire.

#### 2.2.1. BMI and WC Determination

Calculation of BMI was done as weight (kg)/height squared (m^2^). Also, WC was measured at the midway point between the lower border of the lowest rib and the uppermost aspect of the iliac crest. Measurements of BMI, WC, and height were obtained by standardized protocols during physical assessments.

#### 2.2.2. Blood Pressure Measurement

In the right arm, arterial blood pressure was measured in the supine position. Three readings were taken at 5 min intervals, and their means were taken as the final reading.

#### 2.2.3. Assessment of MS Co-Morbidities

MS was diagnosed per the National Cholesterol Education Program’s Adult Treatment Panel III report [10]. Subjects were classified as having MS if demonstrating a minimum three of five criteria: (1) central obesity: WC ≥ 102 cm in males; (2) hypertriglyceridemia: serum TGs ≥ 150 mg/dL; (3) low serum HD L < 40 mg/dL in males; (4) hypertension: systolic blood pressure ≥ 130 mmHg or diastolic blood pressure ≥ 85 mmHg; and (5) hyperglycemia: fasting blood glucose (FBG) ≥ 100 mg/dL.

### 2.3. Biochemical Parameters

In preparation for blood collection, participants fasted for 12 h before undergoing a venous blood draw. In total, 10 mL of venous blood was obtained through a sterile vein puncture in a completely aseptic environment. The blood samples were divided into four portions as follows:

- In total, 1 mL was collected in sodium fluoride/potassium oxalate tubes for the estimation of FBG in plasma using the glucose oxidase method.
- A total of 3 mL was collected in an EDTA tube, centrifuged at 3500 rpm for a duration of 10 min at 4 °C, and then the obtained plasma was stored at −80 °C until further analysis of the residues of OPPs.
- An amount of 4 mL was collected in plain tubes, left to stand for 20 min to allow clot retraction. Then, this was centrifuged for 10 min to withdraw the sera that were stored at −20 °C for the subsequent analysis of cholinesterase activity, insulin, lipid profile parameters, MDA, GGT, ferritin, SOD, TNF-α, and hs-CRP.
- Finally, 2 mL of peripheral whole blood was collected into EDTA-containing tubes for total RNA extraction and gene expression analysis of *PPARγ* and *PON1* by quantitative real-time PCR (qRT-PCR).

#### 2.3.1. Determination of OPP Residues in Plasma

Plasma samples from both groups were analyzed for OPP residues using gas chromatography–mass spectrometry (GC-MS, Agilent 5975C, Agilent Technologies, Santa Clara, CA, USA). The tested OPPs were guided by the history obtained from the farm workers, crop “cotton”, empty packages, and the pesticides found in the stocks. Sample preparation was performed by liquid–liquid extraction using hexane. Briefly, plasma samples were mixed with 1 mL hexane, vortexed, and centrifuged, after which the organic layer was collected for GC–MS analysis.

Chromatographic separation was mediated using a DB-5ms capillary column (30 m × 0.25 mm × 0.25 µm, Agilent Technologies, Santa Clara, CA, USA). The temperature program of the oven ranged from 90 °C to 280 °C, with helium as carrier gas, used at a 1 mL/min flow rate in splitless injection mode. The total run time was 36.8 min.

Calibration and quality control procedures were performed using pesticide-free plasma samples spiked with known concentrations of chlorpyrifos, profenofos, malathion, and dimethoate, with azobenzene used as the internal standard. The quantification depends on the ratio of analyte peak area to internal standard peak area.

Limits of detection (LOD) as well as quantification (LOQ) were determined using fortified blank plasma samples. The LOD was defined as a signal-to-noise ratio of 3:1, while the LOQ was defined as a signal-to-noise ratio of 10:1 with acceptable analytical precision and accuracy. No interfering peaks were noted in blank samples.

The analytical procedure was carried out according to Pérez et al. [11].

Measurement of cholinesterase activity.

Erythrocyte acetylcholinesterase (AChE) and serum butyrylcholinesterase (BChE) activities were determined as markers of chronic OPPs exposure using Ellman’s method [12]. Cholinesterases (AChE and BChE) were assessed using the Cholinesterase Activity Assay Kit (Sigma-Aldrich, Merck, Catalog No: MAK324, St. Louis, MO, USA), following the manufacturer’s instructions, where increased absorbance secondary to 5-thio-2-nitrobenzoic acid formation was observed at 412 nm. Enzyme activity was expressed as units per liter (U/L).

#### 2.3.2. Assessment of FBG, Insulin, and HOMA-IR

Fasting glucose was determined in plasma using the glucose oxidase method (Spinreact, Spain), based on the procedure described by Burrin and Price [13]. Serum insulin (fasting) was determined by using a human insulin ELISA kit (Biosource Europe S.A., Nivelles, Belgium, Cat. No. KAP1251).

IR was evaluated using the Homeostasis Model Assessment of Insulin Resistance (HOMA-IR) according to Matthews et al. [14]:

$$HOM\text{A-}IR=\frac{Fasting\;insulin\;(\mu U/mL)\;\times \;Fasting\;plasma\;glucose\;(mmol/L)}{22.5}$$

Participants with HOMA-IR values above the selected cutoff value were considered insulin resistant. Individuals were deemed insulin resistant if HOMA exceeded 1.64 [14].

#### 2.3.3. Lipid Profile Assessment

The serum lipid profile was analyzed through colorimetric determination using specific assay kits. Total cholesterol was determined as per Allain et al. [15] using the Total Cholesterol Assay Kit (Abcam, Cambridge, UK, ab65390). HDL-cholesterol was determined following Burstein et al. [16], using the HDL and LDL/VLDL Quantification Kit (Abcam, Cambridge, UK, ab204718). Triglycerides (TGs) were quantified using the Triglyceride Assay Kit (Abcam, Cambridge, UK, ab65336) as described by Fossati and Prencipe [17]. LDL-cholesterol was calculated using the Friedewald equation [18]: LDL = TC − HDL − TG/5 (where all lipid concentrations were presented in mg/dL).

#### 2.3.4. Assessment of Oxidative Stress Biomarkers

Serum MDA levels were measured following the method of thiobarbituric acid reactive substances [19] with a Biodiagnostic kit (Cat. No. MD 2529, Biodiagnostic, Giza, Egypt). SOD activity in serum was detected following Marklund and Marklund [20], using a Biodiagnostic kit (Cat. No. SD 2521, Biodiagnostic, Giza, Egypt). Serum GGT levels were measured calorimetrically using Spinreact kits (Sant Esteve de Bas, Girona, Spain or equivalent) [21]. Serum ferritin was measured by a Human Ferritin (FE) ELISA Kit (Cat. No. In-Hu3523, BioVantion, Beijing, China) according to the manufacturer’s instructions) as per the manufacturer’s instructions.

#### 2.3.5. Measurement of Serum TNF-α and hs-CRP Levels

Serum TNF-α levels were measured using an ELISA kit (Sun Red Biotechnology, Shanghai, China, Cat. No. 201-12-0083). Serum hs-CRP was assessed using a Human hs-CRP ELISA Kit (Biosource Europe S.A., Nivelles, Belgium). All protocols were carried out in adherence to the manufacturer’s instructions.

#### 2.3.6. Gene Expression Analysis of *PPARγ* and *PON1*

Following instructions in the manufacturer’s protocol, RNA extraction was accomplished using TRIzol Reagent (Invitrogen, Carlsbad, CA, USA) and quantified spectrophotometrically. RNA purity and integrity were assessed at a 260/280 nm ratio. Subsequently, RNA was reverse transcribed to complementary DNA (cDNA) using the QuantiTect Reverse Transcription Kit (Qiagen, Hilden, Germany, catalog no. 204243). The cDNA was stored at −20 °C until further analysis. The Stratagene Mx3005P qPCR system (Agilent Technologies, Santa Clara, CA, USA) was used to evaluate the mRNA expression of *PPARγ* and *PON1* via quantitative real-time PCR (qRT-PCR). Reactions were conducted in a 20 μL volume including 50 ng of cDNA, 10 pmol/μL of each primer (1 μL each), and 10 μL of SYBR Green 2x Master Mix (Applied Biosystems, Foster City, CA, USA). Amplification commenced with enzyme activation at 95 °C for 15 min, followed by 40 cycles of 95 °C for 15 s and 60 °C for 1 min. mRNA levels were standardized to the transcription level of glyceraldehyde-3-phosphate dehydrogenase (GAPDH) as the internal reference gene, using the ΔΔCT technique, with fold changes computed using the $2^{-\Delta \Delta C_{T}}$ equation. The primers used for real-time PCR are detailed in Table 1.

#### 2.3.7. Statistical Analysis

Analysis of data was done using the software SPSS (Statistical Package for the Social Sciences) version 27. Qualitative variables were presented using their absolute frequencies and compared by chi-square (χ^2^), while quantitative data were presented using means and standard deviations. Student’s *t*-test was applied to compare the means of the two groups. A one-way ANOVA was used to compare means between groups. Correlations between parameters were investigated using Spearman correlation analysis. Also, multivariate linear regression analysis was conducted to identify the risk factors of outcomes. A *p*-value < 0.05 was considered statistically significant, and <0.001 was considered highly statistically significant.

## 3. Results

### 3.1. Assessment of Socio-Demographic, Anthropometric, Occupational, and Smoking Data in Study Groups

Regarding age, no statistically significant difference was observed between the two groups. The mean BMI values were 23 ± 5.09 and 28  ±  6.98, respectively, indicating a highly significant difference between groups (*p* < 0.001). The WC among OPP occupationally exposed workers was statistically higher than in the non-exposed group (106 ± 35 versus 99 ± 22 cm). In total, 42% of the occupationally exposed group had high blood pressure versus 13% of the non-exposed group. The mean duration of pesticide exposure among the occupationally exposed group was 18 ± 4.7 years, and the average hours of OPP spray were 11 ± 2.36 h/week. No significant difference was detected between the two groups regarding smoking, while most of the workers did not use the precautionary equipment (Table 2).

### 3.2. Markers of OPP Exposure in Study Groups

GC-MS of plasma samples revealed the presence of the residues of one or more of the OPPs: chlorpyrifos, malathion, profenofos, and dimethoate in most workers chronically exposed to OPPs. In contrast, residues in the control samples were undetectable (Table 3). As regards the cholinergic enzymes, AChE and BChE were significantly lower in the occupationally exposed group compared to the non-exposed group, *p* < 0.001 (Table 4).

### 3.3. The Impact of OPP Occupational Exposure on Glycemic Parameters and Lipid Profile

**Figure 1:**
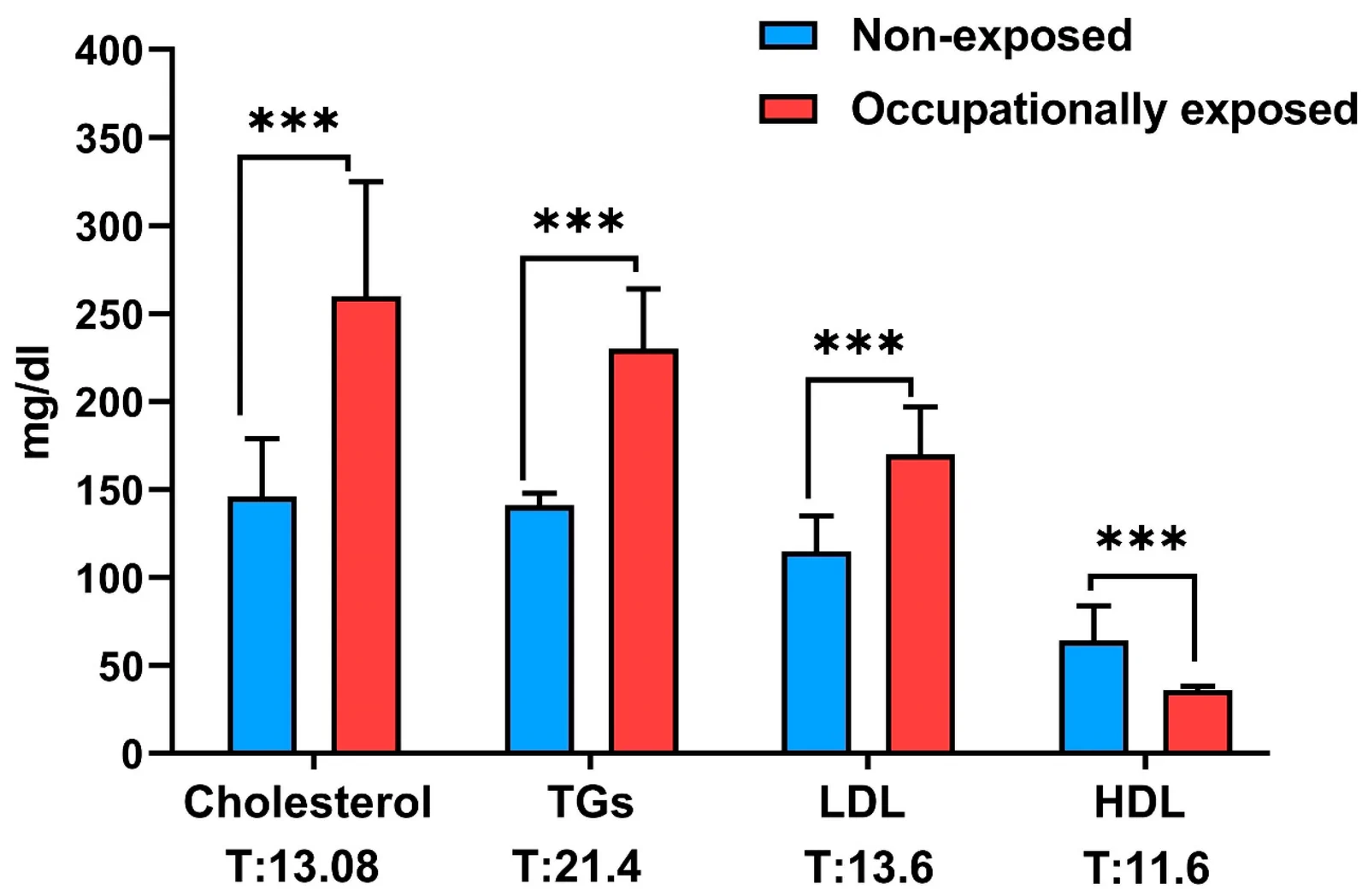
Serum lipid profile in the studied groups. *** High significance is considered when *p* < 0.001, using a *t*-test.

As for glycemic parameters, glucose, insulin, and HOMA-IR levels were statistically significantly higher in the occupationally exposed group compared to the non-exposed, *p* < 0.001 (Table 5). As regards lipid profile, exposed workers had statistically significantly higher levels of total cholesterol, TGs, and LDL than the non-exposed group, with lower HDL levels, *p* < 0.001 (Figure 1).

**Figure 2:**
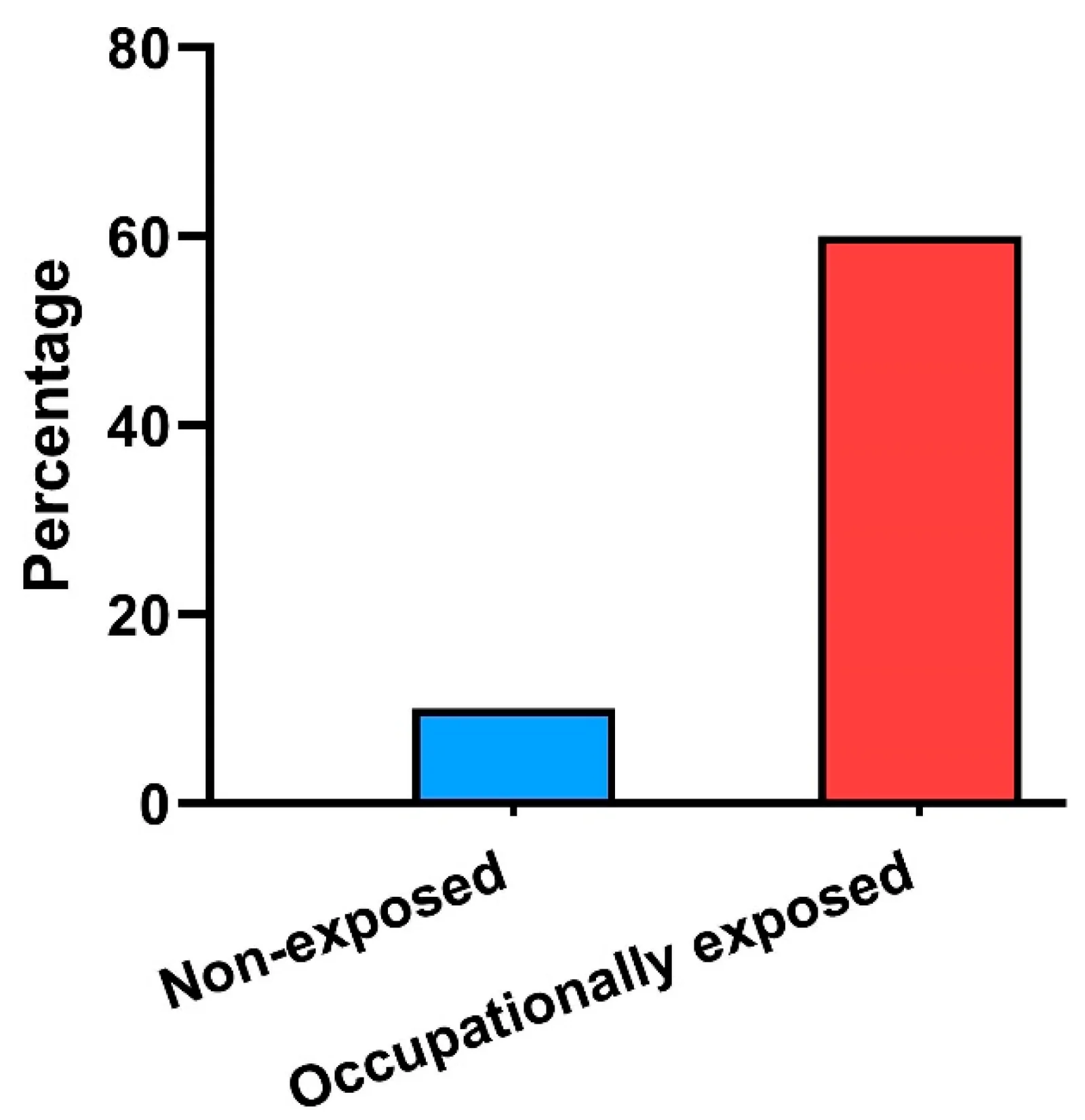
Percentage of metabolic syndrome in the studied groups.

Based on both anthropometric data and biochemical parameters, and following the National Cholesterol Education Program’s Adult Treatment Panel III report, 60% of occupationally exposed workers met the criteria of MS versus 10% of the non-exposed group (Figure 2).

### 3.4. The Impact of OPP Occupational Exposure on Oxidative Stress and Inflammatory Markers

The current results showed that levels of MDA, GGT, and ferritin were significantly higher among exposed workers compared to non-exposed subjects, and exposed workers with MS had statistically significantly higher levels of MDA, GGT, and ferritin than exposed workers without MS, with lower levels of SOD (*p* < 0.001). Average levels of the inflammatory markers (TNF and hs-CRP) were statistically significantly higher in the exposed group compared to the non-exposed group, and among exposed individuals with MS than those without MS (*p* < 0.001) (Table 6).

### 3.5. The Impact of OPP Occupational Exposure on PPARγ and PON1 mRNA Expression

**Figure 3:**
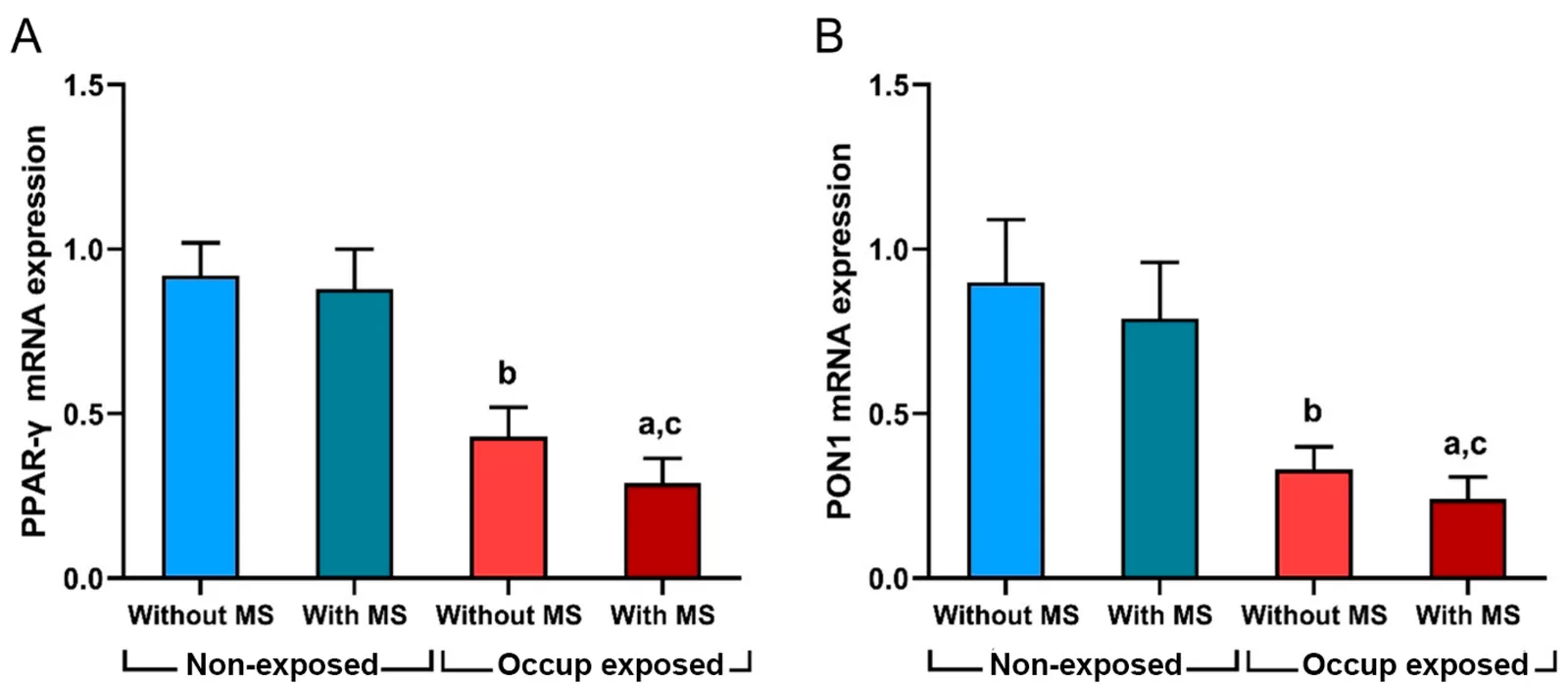
(**A**). *PPARγ* and mRNA expression levels in the studied groups. (**B**). *PON1* and mRNA expression levels in the studied groups. ^a^ *p* < 0.001 when values of those occupationally exposed with MS are copared to the non-exposed group, ^b^ *p* < 0.001 when values of those occupationally exposed without MS are compared to the non-exposed group, ^c^ *p* < 0.001 when values of those exposed with MS are compared to values of the exposed without MS group.

As presented in Figure 3A,B, the *PPARγ* and *PON1* mRNA expressions were significantly lower in exposed farm workers than in non-exposed. Additionally, exposed workers with MS demonstrated a highly significantly lower expression of *PPARγ* and *PON1* mRNA than exposed workers without MS (*p* < 0.001).

### 3.6. Correlation Analysis

In the current study, *PPARγ* gene expression showed a significant positive correlation with HDL, *PON1* expression and SOD, and a negative correlation with oxidative and inflammatory indices, as well as glucose, HOMA-IR, TGs, LDL levels, MDA (nmol/mL), ferritin (ng/mL), GGT (IU/L), TNF-α (pg/mL) and hs-CRP (mg/L) (Table 7).

A correlation between the period of OPP exposure and hours of spraying across various parameters is shown in Table 8. As years of use and hours of spraying increase, HDL, SOD, *PPARγ*, and *PON1* mRNA expression levels significantly decrease, while glucose, HOMA-IR, triglycerides, LDL, MDA, ferritin, GGT, TNF, and hs-CRP significantly increase. Additionally, more hours of OPP spraying are associated with significant increases in MDA, ferritin, TNF, and hs-CRP.

Multiple linear regression analyses showed that longer duration of occupational OPP exposure and greater weekly spraying hours were independently associated with higher HOMA-IR and LDL levels. Greater weekly spraying hours were also associated with higher MDA levels (Table 9). For inflammatory and gene-expression outcomes as shown in Table 10, duration of exposure and spraying hours were positively associated with TNF-α levels and negatively associated with *PON1* and *PPARγ* expression. BMI, WC, blood pressure, and smoking were not independently associated with these outcomes after adjustment for the other variables included in the models.

## 4. Discussion

The prevalence of MS represents a significant global health issue, affecting nearly a quarter of adults worldwide [22]. Former studies suggested that OPPs may contribute to the development of MS by increasing oxidative stress, enhancing inflammatory response, and modulating the expression of the key genes of metabolic regulation [2,23].

Despite the international documentation of the MS context and its potential connection to OPPs, studies on Egyptian farm workers are scarce. So, this study aimed to investigate the probable association between chronic OPP exposure and MS among farm workers in Sharkia Governorate, Egypt, and to assess the potential role of *PPARγ*.

A questionnaire was used for collecting data on the type, duration, and intensity of OPP exposure, safety equipment use, and smoking. Most of the exposed workers had been engaged in cotton farming for more than 15 years, during which they were exposed to one or more OPPs and did not strictly use protective equipment. This behavior is likely related to their limited education and insufficient training [24].

To confirm chronic exposure to OPPs, cholinesterase levels were measured as indicators of such exposure [25]. Furthermore, the plasma residues of the four OPPs (chlorpyrifos, malathion, profenofos, and dimethoate) reported by farm workers were assessed. Most workers had residues of more than one of the tested pesticides, and these levels exceeded the acceptable daily intake (ADI). The presence of OPP residues is believed to increase toxic exposure and complications [24].

According to our results, an overt prevalence of MS was detected in farm workers who minimally exhibited three of the MS comorbidities, including hyperglycemia, IR, obesity, hypertension, and dyslipidemia. Also, the oxidative and inflammatory indices were significantly altered, with substantial downregulation of the mRNA expression of *PPARγ* and *PON1* genes in the exposed group.

The nuclear receptor *PPARγ* is primarily involved in regulating lipid metabolism, glucose homeostasis, insulin balance, and maintaining redox stability and anti-inflammatory defense. The mechanism underlying *PPARγ* gene inhibition has been attributed to both oxidative stress and epigenetic modifications [26].

The nucleophilic nature of OPPs allows them to accept electrons, resulting in reactive oxygen species (ROS) generation. Ref. [27], which disrupt cellular homeostasis and impair signaling pathways, including the PPARγ gene [28].

Also, OPPs have been shown to modulate epigenetic regulators such as histone deacetylases and DNA methyltransferases, potentially leading to the suppression of *PPARγ* expression or alterations in its transcriptional activity [29]. This mechanism is significant in disorders like insulin resistance and MS. Furthermore, OPPs activate inflammatory pathways, including the NF-κB pathway, and elevate pro-inflammatory cytokines [30], thereby antagonizing *PPARγ* expression and potentially exacerbating inflammation and obesity-related metabolic disorders [31].

The signaling pathways of *PPARγ* and *PON1* are closely interconnected, with the *PPARγ* gene serving as a key regulator of *PON1* expression [32]. Stimulation of the *PON1* gene protects against oxidative damage by breaking down oxidized lipids [33] and preventing the oxidation of LDL and other molecules, which are hallmarks of MS [34]. *PON1* downregulation is implicated in MS by impairing redox balance, increasing oxidative stress and inflammation, and disrupting lipid metabolism [35].

OPPs have been reported to downregulate *PON1* expression via two proposed mechanisms. The first is driven by *PPARγ* downregulation, which decreases *PON1* synthesis, while the second involves stimulating hepatic cytokines such as interleukin-1 (IL-1), interleukin-6 (IL-6), and TNF-α, which in turn modulate the *PON1* promoter, suppressing its transcription [36].

In our study, reduced *PPARγ* expression was associated with diminished mRNA expression of the *PON1* gene, matching the finding of Kunachowicz et al. [33]., who reported reduced *PON1* levels in conditions of diminished *PPARγ* activity, including MS. This co-reduction may exacerbate oxidative stress, disrupt antioxidant defenses, and increase susceptibility to oxidative damag.

In the same line, the levels of MDA, GGT, and ferritin were higher in the group exposed to OPPs, accompanied by diminished SOD activity, indicating concurrent oxidative stress. Similarly, chronic OPP-induced lipid peroxidation and decreased antioxidants have been previously acknowledged in former studies [27,32,37].

OPPs exacerbate oxidative stress, mostly by generating ROS and suppressing *PPARγ* and *PON1* genes [23]. Additionally, *PPARγ* downregulation has been reported to suppress Nrf2-mediated transcription, leading to diminished expression of antioxidant genes such as SOD and subsequently exacerbating oxidative stress [6].

Both GGT and ferritin are currently considered indicators of combined MS and oxidative stress [38,39]. Elevated GGT levels are strongly associated with MS components, including IR, dyslipidemia, and obesity. Additionally, GGT activity correlates positively with impaired insulin sensitivity, possibly due to its role in glutathione breakdown, which exacerbates oxidative stress, the main contributor to IR and metabolic disturbances [38]. Monroy-Ramirez et al. reported that downregulation of *PPARγ* disrupts normal liver function, leading to increased GGT expression. The mechanism likely involves oxidative stress and inflammation, where reduced *PPARγ* activity triggers GGT upregulation as a compensatory response [40]. Consistently, a negative correlation was detected in the current study between *PPARγ* and GGT levels in farm workers.

In addition, the accumulation of OPPs with long-term exposure enhances a state of low-grade inflammation [41,42], via triggering inflammatory pathways, like NF-κB, and raising inflammatory cytokines [30]. Also, OPPs suppress *PPARγ*, which helps regulate the anti-inflammatory response by inhibiting the NF-κB pathway, and thus may aggravate the inflammatory response [6].

Concurrently, the exposed groups showed higher levels of TNF-α and hs-CRP, indicating an inflammatory response, while demonstrating a negative correlation with *PPARγ*, suggesting a potential role of *PPARγ* in modulating inflammation.

Both TNF-α and CRP have recently been used as markers of combined MS and inflammation [39]. TNF-α is believed to contribute to MS via boosting adiposity, impairing insulin signaling, and inducing oxidative stress [43]. Likewise, CRP has been associated with both obesity and atherosclerosis in patients with MS [44].

Abdel Aziz et al. reported increased TNF-α and IL-6 in workers exposed to OPPs, particularly those who did not wear protective masks [41]. Also, Yaqub et al. reported that all farm workers with long-term exposure to OPPs had higher CRP levels than the non-exposed subjects, implying concurrent low-grade inflammation [42].

*PPARγ* is also considered critical for maintaining glucose and lipid metabolism. Downregulation of *PPARγ* has been linked to impaired glucose uptake, leading to higher blood glucose levels and IR, the hallmarks of type 2 diabetes [45]. In the current work, increased insulin and fasting blood glucose levels were detected in farm workers chronically exposed to OPPs, consistent with earlier studies [1,46,47,48]. Results also indicated a negative correlation between *PPARγ* gene expression and both glucose and HOMA-IR, suggesting a potential mechanistic contribution of *PPARγ* to hyperglycemia and IR in farm workers with chronic OPP exposure.

Furthermore, the detected evidence of IR in farmers aligned with the increases in their BMI and WC, which are cofactors of IR [49]. OPPs help the proliferation of pre-adipocytes and increase the differentiation of white adipose tissue, causing obesity [50,51].

Also, TG and LDL were significantly higher in exposed workers, while negatively correlating with *PPARγ*, indicating impaired lipid metabolism. Downregulation of *PPARγ* has been implicated in dysregulating lipid and fatty acid metabolism, causing increased circulating TGs and free fatty acids [52]. In addition, reduced *PON1* transcription is thought to contribute to the altered lipid metabolism [35]. Similar to our results, diminished *PPARγ* transcription along with lipid accumulation has been detected in workers exposed to deltamethrin, mancozeb, and prochloraz [53].

Most exposed workers demonstrated elevated blood pressure readings. This can be attributed to increased TGs [45], along with oxidative and inflammatory mechanisms that remodel blood vessels and enhance the sympathetic surge [43]. A cross-sectional study in the USA has also indicated a strong positive correlation between systolic and diastolic blood pressure and the urinary metabolites of chlorpyrifos, diazinon, and methyl parathion [54].

In this study, when years of OPP exposure and weekly spraying hours increased, there was a positive correlation with glucose, HOMA-IR, TGs, LDL, MDA, ferritin, GGT, TNF, and hs-CRP, and a negative correlation with *PPARγ*, *PON1*, HDL, and SOD. These findings are consistent with earlier research showing that OPPs-induced metabolic disruptions were time-dependent [55,56].

Also, we performed multivariable regression analyses adjusting for age, BMI, WC, blood pressure, and smoking to evaluate the independent association between occupational OPP exposure (duration and hours of spraying) and outcomes. Longer duration of occupational OPP exposure and greater weekly spraying hours were independently associated with higher HOMA-IR and LDL levels. Greater weekly spraying hours were also associated with higher MDA levels. For inflammatory and gene-expression outcomes, duration of exposure and spraying hours were positively associated with TNF-α levels and negatively associated with *PON1* and *PPARγ* expression. BMI, WC, blood pressure, and smoking were not independently associated with these outcomes after adjustment for the other variables included in the models. These analyses supported the independent contribution of occupational OPP exposure.

This study has several strengths, as occupational exposure to organophosphorus pesticides was confirmed using both plasma pesticide residue analysis by GC-MS and Cholinesterase activity, providing objective exposure assessment rather than relying solely on self-reported exposure. The study comprehensively evaluated metabolic syndrome by combining clinical, biochemical, oxidative stress, inflammatory, and molecular biomarkers, including *PPARγ* and *PON1* gene expression. The use of participants from agricultural communities with long-term occupational exposure enhances the relevance of the findings to populations at high risk of pesticide exposure.

## 5. Limitations

This study has some limitations that should be considered before interpreting the findings. First, the relatively modest sample size and cross-sectional design of this study limit statistical power and the capability to establish causal relationships. Although our results demonstrate significant associations between *PPARγ* downregulation and MS among OPPs-exposed farmers, these associations should not be considered as evidence of causality. Longitudinal cohort studies and in-depth mechanistic investigations are thus warranted to confirm the proposed pathway and clarify the potential relationships between *PPARγ* dysregulation and MS in chronic OPP exposure.

Second, the sample size was prospectively calculated based on the expected difference in serum cholesterol, which was selected as the primary outcome during the study design because reliable estimates for *PPARγ* expression in a comparable population were not available at that time. We acknowledge that calculating the sample size based on *PPARγ* expression would have been preferable.

Third, some potential confounding factors, including socioeconomic status, dietary habits, physical activity, and other lifestyle-related factors, were not evaluated. Thus, future studies containing a wider range of environmental, behavioral, and socioeconomic confounders are needed.

Finally, this study included only male farmers, which may hinder the generalizability of the results. Future research that includes both male and female workers is highly recommended to assess potential sex-related differences and ensure the broader applicability of the results.

## 6. Conclusions

Based on our findings, farm workers exhibited a higher prevalence of MS. Chronic OPP exposure was associated with increased oxidative stress and inflammation, reduced *PPARγ* and *PON1* expression, disturbances in glucose and lipid metabolism, and increased IR. The observed associations between *PPARγ* downregulation, metabolic disturbances, and oxidative and inflammatory markers suggest that *PPARγ* dysregulation may represent a potential mechanistic link between chronic OPP exposure and MS. However, the proposed role of *PPARγ* dysregulation remains a biologically plausible hypothesis and should be interpreted with caution, as it necessitates further validation through longitudinal and functional mechanistic studies. Additionally, targeted educational programs should be provided to improve farm workers’ awareness of the safe handling and usage of OPPs, thereby reducing the MS risk. Also, routine screening for MS-related comorbidities in farm workers is highly recommended for early detection of MS and proper intervention.

## Figures and Tables

**Table 1:** Primer sequences for the *PPARγ* and *PON1* genes.

Gene	Primer	Accession Number	Length pb
*PPARγ*	F: TACTGTCGGTTTCAGAAATGCC R. GTCAGCGGACTCTGGATTCAG	NM_015869.5	120
*PON1*	F: CCTGCAATAATATGAAACAACCTG R: CTAGAACACGAAAAGTGAAAGAAAAC	NM_000446.6	150
*GAPDH*	F: ACAACTTTGGTATCGTGGAAGG R: GCCATCACGCCACAGTTTC	NM_002046.7	170

**Table 2:** Socio-demographic, anthropometric, occupational, and smoking data in study groups.

	Non-Exposed Group *N* = 70 (%)	Occupationally Exposed Group *N* = 70 (%)	χ^2^	*p*-Value
Age				
• <30	41 (58.6)	31 (44.3)		
• ≥30	29 (41.4)	39 (55.7)	2.8	0.09
Mean ± SD	26.9 ± 3.3	27.6 ± 4.8	1.01	0.31
Body Mass Index				
• Normal	35 (50)	10 (14.3)		
• Overweight	16 (22.9)	22 (31.4)	21.16	<0.001 **
• Obese	19 (27.1)	38 (54.3)		
Mean ± SD	23 ± 5.09	28 ± 6.98	4.84	<0.001 **
Waist circumference				
• <102 cm	47 (67.1)	25 (35.7)		
• >102 cm	23 (32.9)	45 (64.3)	13.7	<0.001 **
Mean ± SD	90 ± 22	106 ± 35	3.23	<0.001 **
Blood pressure				
• Low	12 (17.1)	10 (14.3)		
• Normal	45 (64.3)	18 (25.7)	27.4	<0.001 **
• High	13 (18.6)	42 (60)		-
Type of OPPs				
Chlorpyrifos		62 (88%)		
Malathion		34 (48%)		
Profenofos	-	39 (55%)	-	-
dimethoate		13 (18%)		
Duration of the OPPs exposure				
• <15 y	NA	25 (35.7)	-	-
• ≥15 y		45 (64.3)		
Mean ± SD		18 ± 4.7		
Hours of OPP				
spray/week				
• <10 h	NA	30 (42.9)	-	-
• ≥10 h		40 (57.1)		
Mean ± SD		11 ± 2.36		
Precautions equipment				
• Yes	NA	13 (18.6)		
• No		57 (81.4)	-	-
Smoking				
• Yes	26 (37.1)	24 (34.2)		
• No	44 (62.8)	46 (65.7)	0.12	0.72

** A highly statistically significant difference. The *t*-test was used for quantitative data, while the chi-square for qualitative data. NA not applicable.

**Table 3:** Residues of OPPs in the plasma of the exposed group.

OPP	ADI mg/kg	(%)	Average Amount mg/kg
Chloropyriphos mg/kg	0.01	84	0.248
Malathion mg/kg	0.3	43	0.383
Profenofos mg/kg	0.01	51	0.198
Dimethoate mg/kg	0.002	9	0.0023
Combined residues	-	75	-
No	-	16	-

ADI: Acceptable daily intake.

**Table 4:** Cholinergic enzymes in study groups.

	Non-Exposed Group	Occupationally Exposed Group	*T-Test*	*p*-Value
RBC-AChE mU/µmol Hb	0.30 ± 0.09	0.10 ± 0.03	17.6	<0.001 **
BCHE				
µmol/L/min	0.033 ± 0.007	0.018 ± 0.001	17.8	<0.001 **

** A highly statistically significant difference.

**Table 5:** Glycemic parameters of OPPs in study groups.

	Non-Exposed Group	Occupationally Exposed Group	*T-Test*	*p*-Value
FBG (mg/dL)	89.7 ± 9.2	190.7 ± 50.9	16.3	<0.001 **
Insulin (µIU/mL)	4.4 ± 1.85	11.7 ± 2.34	20.5	<0.001 **
HOMA-IR	1.07 ± 0.55	5.9 ± 1.08	33.3	<0.001 **

** A highly statistically significant difference.

**Table 6:** Serum levels of oxidation and inflammatory markers in the study groups.

	Non-Exposed Group		Occupationally Exposed Group	
	Without MS	With MS	Without MS	With MS
SOD (U/mL)	80.2 ± 19.22	73.2 ± 14.02	67.2 ± 13.45 ^b^	50.4 ± 10.09 ^a,c^
MDA (nmol/mL)	2.92 ± 0.98	3.54 ± 0.09	4.43± 1.02 ^b^	6.63 ± 2.01 ^a,c^
Ferritin (ng/mL)	200 ±46.84	250 ±53.12	597.2 ± 97.5 ^b^	920.4 ± 300.7 ^a,c^
GGT (IU/L)	20 ± 5.84	23.70 ±6.84	28 ± 6.041 ^b^	32.9 ± 6.1 ^a,c^
TNF-α (pg/mL)	6.19 ± 3.01	7.51 ± 2.48	12.51 ± 3.09 ^b^	18.51 ± 2.78 ^a,c^
hs-CRP (mg/L)	1.11 ± 0.22	2.82 ± 0.21	3.19 ± 1.01 ^b^	8.1 ± 0.8 ^a,c^

^a^ *p* < 0.001 when values of those exposed with MS are compared to the non-exposed group, ^b^ *p* < 0.001 when values of those exposed without MS are compared to the non-exposed group, ^c^ *p* < 0.001 when values of those exposed with MS are compared to values of exposed without MS group.

**Table 7:** Correlation between *PPARγ* and blood glucose, insulin resistance, lipid parameters, *PON1*, oxidative and inflammatory markers.

	PPAR γ	
	r	*p*-Value
**FBG (mg/dL)**	−0.36	0.02 *
**HOMA-IR**	−0.41	0.001 *
**TGs (mg/dL)**	−0.34	0.05 *
**LDL (mg/dL)**	−0.38	0.007 *
**HDL (mg/dL)**	0.34	0.05 *
***PON1***	0.50	<0.001 **
**MDA (U/mL)**	−0.55	<0.001 **
**SOD (nmol/mL** **)**	0.49	<0.001 **
**Ferritin (ng/mL)**	−0.51	<0.001 **
**GGT (IU/L** **)**	−0.48	<0.001 **
**TNF-α (pg/mL)**	−0.61	<0.001 **
**hs-CRP (mg/L** **)**	−0.50	<0.001 **

* Statistically different. ** A highly statistically significant difference.

**Table 8:** Correlation between duration of OPP exposure and hours of spraying with each of the cholinergic enzymes, glycemic, lipid, oxidative, inflammatory, and genetic parameters.

Duration of Pesticide Exposure			Hours of Pesticide Spray	
	r	*p*-Value	r	*p*-Value
FBG (mg/dL)	0.48	<0.001 **	0.26	0.08
HOMA-IR (µIU/mL)	0.4	0.002 *	0.18	0.17
TGs (mg/dL)	0.41	0.001 *	0.14	0.23
HDL (mg\dL)	−0.33	0.006 *	−0.22	0.11
LDL (mg\dL)	0.44	0 001 *	0.33	0.09
Ferritin (ng/mL)	0.4	0.001 *	0.55	<0.001 **
MDA (nmoL/mL)	0.37	0.001 *	0.58	<0.001 **
SOD (U/mL)	−0.35	0.003 *	−0.40	0.001 *
GGT (IU/L)	0.39	0.001 *	0.34	0.09
TNF-α (pg/mL)	0.37	0.001 *	0.43	<0.001 **
hs-CRP (mg/L)	0.33	0.004 *	0.47	<0.001 **
*PPARγ*	−0.48	<0.001 **	−0.49	<0.001 **
*PON1*	−0.52	<0.001 **	−0.50	<0.001 **

* Statistically different. ** A highly statistically significant difference.

**Table 9:** Multiple linear regression analysis of factors independently associated with HOMA-IR, LDL, and MDA.

Predictor	B	S.E.	β	t	*p*-Value
HOMA-IR					
Duration of exposure	0.27	0.08	0.36	3.38	0.001 *
Hours of spraying/week	0.41	0.14	0.31	2.93	0.005 *
BMI	0.21	0.13	0.24	1.62	0.110
WC	1.21	0.74	0.15	1.64	0.106
Blood pressure	3.08	2.19	0.12	1.41	0.163
Smoking	6.10	3.51	0.18	1.74	0.087
LDL					
Duration of exposure	0.58	0.26	0.29	2.23	0.029 *
Hours of spraying/week	0.64	0.28	0.33	2.29	0.025 *
BMI	1.41	1.17	0.14	1.21	0.231
WC	0.46	0.24	0.24	1.92	0.059
Blood pressure	6.30	4.90	0.18	1.29	0.202
Smoking	8.70	7.10	0.12	1.22	0.227
MDA					
Duration of exposure	2.08	1.05	0.21	1.98	0.052
Hours of spraying/week	11.60	4.60	0.33	2.52	0.014 *
BMI	1.21	0.70	0.15	1.73	0.089
WC	3.10	2.05	0.10	1.51	0.136
Blood pressure	8.10	7.00	0.11	1.16	0.250
Smoking	1.21	0.74	0.15	1.64	0.106

B = unstandardized coefficient; S.E. = standard error; β = standardized coefficient. * Statistically different.

**Table 10:** Multiple linear regression analysis of factors independently associated with TNF-α, *PON1*, and *PPARγ* expression.

Predictor	B	S.E.	β	t	*p*-Value
TNF-α					
Duration of exposure	0.118	0.032	0.25	3.66	<0.001 **
Hours of spraying/week	13.20	1.30	0.74	10.15	<0.001 **
BMI	8.70	7.10	0.12	1.22	0.227
WC	0.46	0.24	0.24	1.92	0.059
Blood pressure	6.30	4.90	0.18	1.29	0.202
Smoking	0.45	0.90	0.68	0.50	0.619
*PON1*					
Duration of exposure	−2.98	1.16	−0.36	−2.57	0.013 *
Hours of spraying/week	−4.15	1.28	−0.31	−3.24	0.002 *
BMI	0.23	0.14	0.15	1.60	0.115
WC	0.45	0.90	0.68	0.50	0.619
Blood pressure	8.70	7.10	0.12	1.22	0.227
Smoking	1.21	0.74	0.15	1.64	0.106
*PPARγ*					
Duration of exposure	−0.62	0.18	−0.34	−3.44	0.001 *
Hours of spraying/week	−0.27	0.08	−0.36	−3.38	0.001 *
BMI	0.21	0.31	0.24	0.68	0.499
WC	1.21	0.74	0.15	1.64	0.106
Blood pressure	6.10	3.51	0.18	1.74	0.087
Smoking	0.24	0.14	0.17	1.71	0.092

B = unstandardized coefficient; S.E. = standard error; β = standardized coefficient. * Statistically different. ** Highly statistically different.

## Data Availability

The datasets used and/or analyzed during the current study are available from the corresponding author on reasonable request.
